# In Vitro and Clinical Evaluations of the Drug-Drug Interaction Potential of a Metabotropic Glutamate 2/3 Receptor Agonist Prodrug with Intestinal Peptide Transporter 1[Fn FN3]

**DOI:** 10.1124/dmd.116.071118

**Published:** 2017-02

**Authors:** Y. Anne Pak, Amanda J. Long, William F. Annes, Jennifer W. Witcher, Mary Pat Knadler, Mosun A. Ayan-Oshodi, Malcolm I. Mitchell, Phillip Leese, Kathleen M. Hillgren

**Affiliations:** Eli Lilly and Company, Lilly Corporate Center, Indianapolis, Indiana (Y.A.P., A.J.L.,W.F.A., J.W.W., M.P.K., M.A.A.-O., M.I.M., K.M.H.); and Quintiles Transnational, Clinical Pharmacology, Overland Park, Kansas (P.L.)

## Abstract

Despite peptide transporter 1 (PEPT1) being responsible for the bioavailability for a variety of drugs, there has been little study of its potential involvement in drug-drug interactions. Pomaglumetad methionil, a metabotropic glutamate 2/3 receptor agonist prodrug, utilizes PEPT1 to enhance absorption and bioavailability. In vitro studies were conducted to guide the decision to conduct a clinical drug interaction study and to inform the clinical study design. In vitro investigations determined the prodrug (LY2140023 monohydrate) is a substrate of PEPT1 with *K*_m_ value of approximately 30 *µ*M, whereas the active moiety (LY404039) is not a PEPT1 substrate. In addition, among the eight known PEPT1 substrates evaluated in vitro, valacyclovir was the most potent inhibitor (IC_50_ = 0.46 mM) of PEPT1-mediated uptake of the prodrug. Therefore, a clinical drug interaction study was conducted to evaluate the potential interaction between the prodrug and valacyclovir in healthy subjects. No effect of coadministration was observed on the pharmacokinetics of the prodrug, valacyclovir, or either of their active moieties. Although in vitro studies showed potential for the prodrug and valacyclovir interaction via PEPT1, an in vivo study showed no interaction between these two drugs. PEPT1 does not appear to easily saturate because of its high capacity and expression in the intestine. Thus, a clinical interaction at PEPT1 is unlikely even with a compound with high affinity for the transporter.

## Introduction

**Fig. 1.**
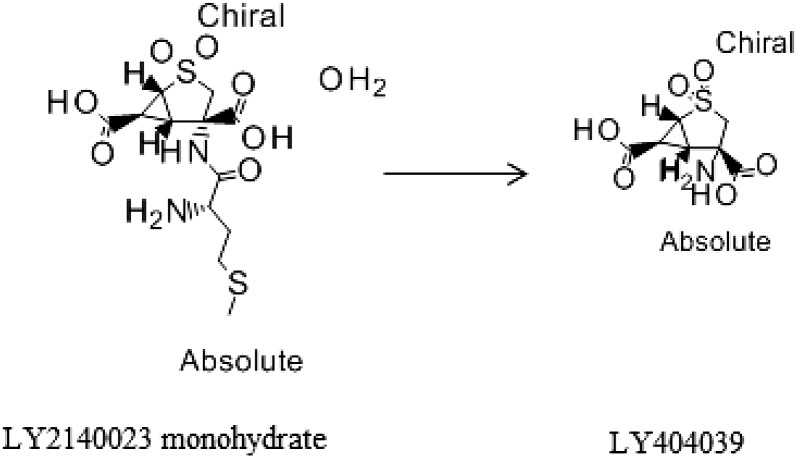
Conversion of LY2140023 monohydrate to LY404039.

Pomaglumetad methionil (LY2140023 monohydrate), a potent and selective methionil prodrug of the metabotropic glutamate 2/3 (mGlu2/3) receptor agonist LY404039 (the active moiety) (Monn et al., 2005), was developed to use the intestinal peptide transporter 1 (PEPT1; SLC15A1) to enhance the bioavailability of the active moiety (Patil et al., 2007). The orally administered prodrug is rapidly absorbed in the intestine and hydrolyzed to the active moiety (Fig. 1) with no further known metabolism (Moulton et al., 2015). PEPT1 is highly expressed at the apical membrane of enterocytes in the small intestine and uses the inwardly directed proton gradient to move substrates across the cell membrane (Daniel and Kottra, 2004; Brandsch et al., 2008). This inwardly directed proton gradient is maintained by an acidic microenviroment in intestinal epithelia. The intervillous pH at the intestinal epithelia ranges from 6.1 to 6.6, whereas intracellular pH of enterocyte is approximately 7.3 (Brandsch et al., 2008). In addition to di- and tri-peptides, PEPT1 has been shown to transport a variety of chemically diverse compounds such as angiotensin converting enzyme inhibitors (captopril and enalapril), *β*-lactam antibiotics (cefadroxil and cephalexin), and valacyclovir, the prodrug of acyclovir. Given its broad substrate specificity and high capacity, targeting PEPT1 has become a strategy in drug development for improving poor intestinal absorption. For example, acyclovir bioavailability was significantly increased after the introduction of an ester linkage to valine resulting in the PEPT1 substrate, valacyclovir (Ganapathy et al., 1998).

PEPT1 is highly expressed in the intestines, brain, and kidney. Within the intestines, PEPT1 expression is localized in the duodenum, ilieum, and jejunum of mice (Groneberg et al., 2001; Jappar et al., 2010) and humans (Walker et al., 1998; Groneberg et al., 2001). Recent proteomic data showed that PEPT1 is the most abundantly expressed transporter in the intestines (Drozdzik et al., 2014), with higher expression than apical uptake transporters such as organic anion transporting polypeptide 2B1 (OATP2B1/SLCO2B1) and apical sodium/bile acid cotransporter (ASBT/SLC10A2), and apical efflux transporters such as P-glycoprotein/ABCB1, breast cancer resistance protein (BCRP/ABCG2), and the multidrug resistance-associated proteins (MRPs/ABCCs).

Because PEPT1 is considered to be a low-affinity and high-capacity transporter with *K*_m_ values ranging typically from high micromolar to low milimolar for most of marketed PEPT1 substrates, there is no major concern of an interaction between drugs transported by PEPT1 during absorption (Hillgren et al., 2013). Phan and colleagues (2003) observed no statistically different changes in acyclovir AUC when valacyclovir was administered alone or co-dosed with cephalexin. However, potential for interaction cannot be ruled out if a high-affinity PEPT1 substrate is coadminstered with a strong PEPT1 inhibitor. In this study, we determined LY2140023 (the prodrug) to be a high-affinity PEPT1 substrate and evaluated its interaction potential with a strong PEPT1 inhibitor, valacyclovir, using an in vitro system. A clinical drug-drug interaction (DDI) study in healthy subjects was then conducted to determine if there was an interaction observed in vivo when the prodrug and valacyclovir were coadministered.

## Materials and Methods

### In Vitro Methods

#### Materials.

LY2140023, LY404039, [^14^C]LY2140023, and [^14^C]LY404039 were synthesized by Eli Lilly and Company (Indianapolis, IN). The purity of radiolabeled compounds were 97% and higher. Cefadroxil, cephalexin, enalapril, captopril, 5-aminolevulinic acid (ALA), glycylsarcosine (Gly-Sar), and triton X-100 were purchased from Sigma (St. Louis, MO); l-DOPA was acquired from Isotec (Miamisburg, OH). Valacyclovir and [^14^C]Gly-Sar were obtained from Moravek Biochemical Inc. (Brea, CA). Cell Culture reagents were purchased from Invitrogen (Carlsbad, CA). All reagents were highest commercial grade, stored properly, and used before expiration date.

#### Cell Culture.

HeLa cells were obtained from American Type Culture Collection (Manassas, VA). Cells were passaged and transiently transfected as described previously (Zhang et al., 2004) with minor modifications. Briefly, cells were seeded at a density of 65,000 cells/ml in 24-well plates. After achieving 50 to 80% confluency, cells were transfected with SLC15A1, gene for PEPT1 (accession number NM_005073) inserted in pcDNA3.1 or pcDNA3.1 empty vector (Invitrogen) using Fugene 6 (Roche, Indianapolis, IN) following the manufacturer’s protocol.

#### System Validation.

Assessment of both the proton-dependent uptake (Liang et al., 1995) and the concentration-dependent uptake of [^14^C]Gly-Sar by the transiently transfected HeLa cells was used to validate the in vitro system. For proton-dependent uptake, [^14^C]Gly-Sar was measured 24 hour post transfection at either pH 6.0 or 7.5. PEPT1- and empty vector-transfected cells were incubated with [^14^C]Gly-Sar (50 *µ*M) for 5 minutes at room temperature. Cells transfected with empty pcDNA3.1 vector were used to measure the passive diffusion of [^14^C]Gly-Sar. For concentration-dependent uptake, transfected cells were incubated with [^14^C]Gly-Sar at concentrations from 50 to 5000 *µ*M for 3 minutes at room temperature. For both proton- and concentration-dependent experiments, cells were washed three times with ice-cold phosphate-buffered saline after incubation with Gly-Sar to stop uptake and then lysed with 0.3 ml 1% triton X-100 in phosphate-buffered saline for 30 minutes at room temperature. Scintillation counting was conducted to determine radioactivity, and protein levels were determined by the bicinchoninic acid method (Smith et al., 1985).

#### Proton- and Time-Dependent Transport of the Prodrug and its Active Moiety.

The PEPT1 mediated [^14^C]LY2140023 (30 *μ*M) and [^14^C]LY404039 (30 *μ*M) uptake was measured in cells 24 hours post transfection. The time-dependent transport of LY2140023 and LY404039 was conducted using buffers prepared at either pH 6.0 or 7.5 as described above. The cells transfected with empty pcDNA3.1 vector were used to measure the passive diffusion of LY2140023 and LY404039. The cells were incubated for 1, 2.5, 5, 7.5, 10, and 15 minutes at room temperature and were washed, lysed, and contents quantified as described above.

#### Concentration-Dependent Uptake to Measure Kinetic Parameters of the Prodrug.

The concentration dependent uptake of [^14^C]LY2140023, ranging from 5 up to 149 *μ*M, was determined in PEPT1 transfected HeLa cells with 2- to 3-minute incubation at room temperature. The mean passive diffusion of prodrug at each concentration was obtained in parallel experiments in HeLa cells transfected with pcDNA3.1 empty vector and subtracted from the uptake mediated by PEPT1. The corrected data were fitted by WinNonlin Professional, version 5.0.1 or 5.3 (Certara, L.P., St. Louis, MO). The kinetic parameters of the prodrug mediated by PEPT1 were estimated by utilizing the following equation:
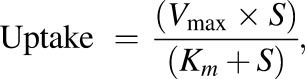
where S is the substrate concentration (*μ*M), *V*_max_ is the maximum uptake rate (pmol/min/mg), and *K*_m_ is the concentration where uptake reaches a half of *V*_max_ (*μ*M).

#### Inhibition.

An inhibition assay was conducted using 25 *μ*M [^14^C]Gly-Sar (0.278 *μ*Ci/ml) as a probe substrate to assess if the uptake of [^14^C]Gly-Sar was inhibited by prodrug (5 to 1000 *μ*M). The cells were incubated for 3 minutes in uptake buffer containing 25 *μ*M [^14^C]Gly-Sar and different concentrations of LY2140023 at room temperature. The uptake was corrected for background determined by parallel experiment in cells transfected with pcDNA3.1 empty vector. Cell lysing and protein quantification were performed as described above.

Seven previously reported PEPT1 substrates (Zhang et al., 2004) and Gly-Sar, a PEPT1 probe substrate, were also assessed to determine their inhibitory potential on 10 *μ*M [^14^C]LY2140023 (0.193 *μ*Ci/ml) uptake mediated by PEPT1. Two separate inhibition studies were conducted as described above using ALA (0.1 to 5 mM or 0.25 to 5 mM), captopril (0.5 to 40 mM), cefadroxil (0.3 to 10 mM), cephalexin (1 to 40 mM), enalapril (1 to 15 or 1 to 20 mM), l-DOPA (2.5 to 25 mM), Gly-Sar (0.1 to 5 or 0.25 to 5 mM), and valacyclovir (0.1 to 5 mM). The solubility of compounds at these concentrations was checked by using a fiber-optic light source. For L-DOPA, the concentration range for inhibition was limited by the solubility. Less than 50% inhibition of [^14^C]LY2140023 uptake was reached by 25 mM L-DOPA, thus IC_50_ was estimated using the inhibition of Gly-Sar at 20 mM as the complete inhibition of PEPT1 (complete inhibition was assumed at 20 mM, because IC_50_ value of Gly-Sar was 0.99 mM).

The calculation of the concentration of inhibitor resulting in 50% inhibition (IC_50_ value) was then determined by nonlinear regression analysis using WinNonlin Professional, version 5.0.1, where:


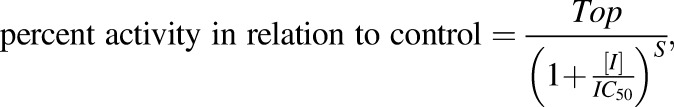


where [I] is an inhibitor concentration in micromoles. Top is the highest % activity in the absence of inhibitor, and S is the slope factor.

### Clinical Study

The study (H8Y-MC-HBCF) was conducted at the Quintiles Research Unit (Overland Park, KS) in accordance with applicable laws, good clinical practice, and the Declaration of Helsinki. The protocol and consent forms were approved by the MidLands Institutional Review Board, L.C.C.C. (Overland Park, KS).

The study was conducted as a three-period, fixed-sequence design in which subjects received a single dose of 1000 mg valacyclovir (Period 1), a single dose of 80 mg of LY2140023 (Period 2), and then a dose of 80 mg of LY2140023 coadministered with 1000 mg valacyclovir (Period 3); all periods were separated by a 5- to 10-day washout. Serial blood samples were collected for assessment of valacyclovir, acyclovir (refer to Ganapathy et al., 1998 for structure of valacyclovir and acyclovir), LY2140023, and LY404039 pharmacokinetics (PK). Urine was collected from 0 to 6, 6 to 12, and 12 to 24 hours postdose for analysis of valacyclovir, acyclovir, and/or the active moiety in the urine. Safety was assessed by collection of adverse events, clinical laboratory evaluations, electrocardiograms, and neurologic examinations.

Eligible subjects were comprised of healthy men and women between 18 and 65 years of age, inclusive, with a body mass index between 19 and 32 kg/m^2^, inclusive. Subjects unable to cease use of xanthines, cigarettes, or over-the-counter or prescription medication for the duration of the trial were excluded. All subjects signed written informed consent before participation in the study.

A sufficient number of subjects were enrolled to obtain 18 subjects to complete the study. This sample size was to provide at least 90% power to show the inclusion of the 90% confidence intervals (CI) of the ratio of area under the curve (AUC) geometric means between the test (LY2140023 + valacyclovir) and reference (LY2140023 alone) fall within the interval (0.80, 1.25).

#### Bioanalysis.

Plasma samples were analyzed for valacyclovir, acyclovir, LY2140023 (prodrug), and LY404039 (active moiety) using validated turbo ion spray liquid chromatography/tandem mass spectrometric methods. For the prodrug and active moiety, the lower limit of quantification (LLQ) was 0.25 ng/ml and the upper limit of quantification (ULQ) was 100 ng/ml for both analytes (Annes et al., 2015). For valacyclovir and acyclovir, the LLQ was 100 ng/ml and the ULQ was 1000 ng/ml for both analytes.

Urine samples were analyzed for valacyclovir, acyclovir, and/or active moiety using a validated liquid chromatography/tandem mass spectrometric method. No analysis of the prodrug was performed because previous studies have shown that it is not excreted in the urine. For the active moiety, the LLQ was 50 ng/ml and the ULQ was 5000 ng/ml. For valacyclovir and acyclovir, the LLQ was 100 ng/ml and the ULQ was 20,000 ng/ml.

For all bioanalytical methods, samples above the limit of quantification were diluted and reanalyzed to yield results within the calibrated range.

#### Pharmacokinetic Analyses.

Plasma concentration-time data for valacyclovir, acyclovir, LY2140023, and LY404039 were analyzed by standard noncompartmental methods of analysis using WinNonlin Version 5.3. Actual sampling times were used in the analyses with the exception of predose times, which were set to 0 hour. Area under the curve (AUC) values were determined using log-linear trapezoid methods. When calculating CL/F and Vz/F for the active moiety, the dose of the prodrug was adjusted based on the molar ratio of active moiety to prodrug (0.64).

Urine concentration and volume data were measured for LY404039, valacyclovir, and acyclovir. Amounts excreted over each collection interval were summed to determine the cumulative amount excreted over the 24-hour collection interval [Ae(0–24)]. The fraction of the dose excreted (fe) was also determined. For the active moiety dose, the 0.64 correction factor was used as described previously. Similarly, for acyclovir, the valacyclovir dose was adjusted based on the molar ratio of acyclovir to valacyclovir (0.694). Apparent renal clearance was estimated using the cumulative amount excreted up to the last collection interval and plasma AUC(0–24).

Although PK parameters were determined for all subjects with concentration-time data, if vomiting occurred within 5 hours postdose the concentration-time data and PK parameters from that dosing period were not included in any data summaries or statistical analysis. Only one subject (in Period 2) had PK data excluded because of vomiting.

The primary PK parameters (*C*_max_ and AUC) for LY2140023, LY404039, valacyclovir, and acyclovir were compared when the prodrug and valacyclovir were administered alone and in combination. AUC(0–∞) was used for all analytes except valacyclovir where AUC(0–3) was assessed. Parameters were compared using linear mixed effect model where treatment (80 mg of LY2140023 administered alone, 1000 mg valacyclovir administered alone, and 80 mg of LY2140023 coadministered with 1000 mg valacyclovir) was included as a fixed factor, and subject was a random factor. The parameters were log transformed before analysis. The least squares means (LSM) for each treatment and the 90% confidence intervals (CI) for the difference in means between test and reference treatment groups were estimated from the model and back transformed from the log scale to provide estimates of the geometric means and 90% CIs for the ratio of geometric means. The analysis of t_max_ was based on a nonparametric method. Medians and range for treatments and the *P* value computed for comparison of median values using Wilcoxon signed rank test are presented.

#### Safety.

There were no serious adverse events (AEs) in this study. One subject discontinued from the study after experiencing a mild AE of urticaria that occurred approximately 4 hours after receiving 1000 mg of valacyclovir alone.

Most AEs were mild or moderate; one severe AE of headache occurred after valacyclovir alone. The most common AEs after prodrug alone were nausea, dizziness, somnolence, and headache. The AE profile for LY2140023 coadministered with valacyclovir was similar to LY2140023 alone.

## Results

### In Vitro Results

#### Validation of In Vitro System.

The transport function of PEPT1 in transiently transfected HeLa cells was first assessed using extracellular pH of 6.0 and 7.5 to show that the uptake of the PEPT1 probe substrate [^14^C]Gly-Sar was a proton-dependent process. The transport of [^14^C]Gly-Sar at pH 6.0 was approximately five times higher than at pH 7.5 (Supplemental Fig. S1). Empty pcDNA3.1 vector-transfected cells (control cells) showed negligible passive permeability at both pH 6.0 and 7.5 (Supplemental Fig. S1). The transport kinetics of [^14^C]Gly-Sar were assessed in the concentrations ranges from 50 *μ*M to 5 mM to confirm the transport function of the in vitro system. Subsequently, the data were fitted in a model representing the uptake as a combination of passive diffusion and PEPT1-mediated transport. PEPT1-mediated transport of [^14^C]Gly-Sar exhibited saturable kinetics with *K*_m_ and *V*_max_ values of 427.9 *μ*M and 3975.3 pmol/min/mg, respectively (Supplemental Fig. S2), similar to reported K_m_ and V_max_ of 500 *µ*M and 3517 pmol/min/mg in CHO cells transfected with PEPT1 (Han, 1999). The passive diffusion rate measured in control cells was 0.0972 *μ*l/min/mg.

#### Inhibitory Effect of the Prodrug on PEPT1 [^14^C]Gly-Sar Transport.

The inhibitory effect of LY2140023 on the PEPT1 probe substrate [^14^C]Gly-Sar (25 *μ*M) was examined at concentrations ranging from 5 to 1000 *μ*M. The accumulation of [^14^C]Gly-Sar in the absence of LY2140023 was used as the positive control. The passive diffusion of [^14^C]Gly-Sar at each concentration was measured by conducting parallel experiments in control cells transfected with a pcDNA3.1 empty vector and the values were subtracted from the accumulation in PEPT1-transfected HeLa cells. The estimated IC_50_ values of the prodrug in two separate experiments were 0.023 ± 0.09 and 0.013 ± 0.07 mM, respectively, with the mean value of 0.018 mM (Table 2).

#### Evaluation of Prodrug and Active Moiety Uptake by PEPT1.

**Fig. 2.**
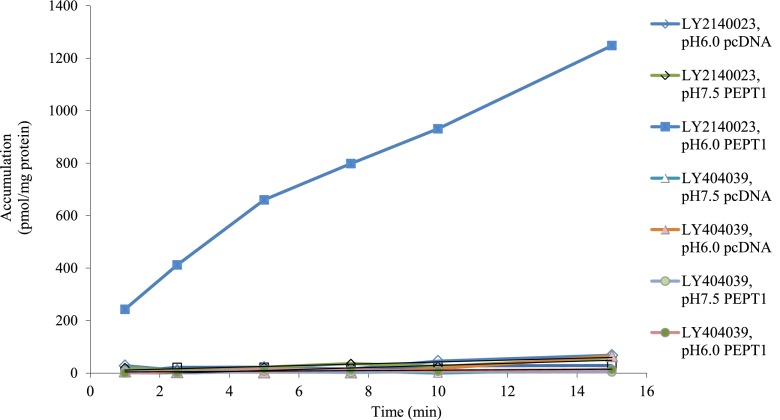
Time- and pH-dependent accumulation of [^14^C]LY2140023 (30 *µ*M) and [^14^C]LY404039 (30 *µ*M). The experiments were conducted using HeLa cells transiently transfected with either PEPT1 or empty vector. The reactions were performed using buffer at either pH 6.0 or 7.5. The cells were incubated with either LY2140023 or LY404039 from 1 to 15 minutes at room temperature. Each symbol represents the mean of two wells per each time point and treatment in 24-well plate.

The uptake of amino-acid prodrug [^14^C]LY2140023 (30 *μ*M; prodrug) or the active drug [^14^C]LY404039 (30 *μ*M; active moiety) was conducted at pH 6.0 or 7.5 in HeLa cells transiently transfected with PEPT1 to determine if LY2140023 or LY404039 were substrates of PEPT1 (Fig. 2). The level of accumulation of the active moiety in HeLa cells transfected with PEPT1 was similar to its passive accumulation, indicating that it was not transported by PEPT1. In contrast, as illustrated in Fig. 2, prodrug uptake was both a proton- and time-dependent process, suggesting that it was a PEPT1 substrate. Although the passive permeability of the prodrug, as indicated by uptake into the pcDNA3.1 empty vector, was slightly higher than that seen for the active moiety it is still negligible compared with the pH-dependent transport mediated by PEPT1.

**Fig. 3.**
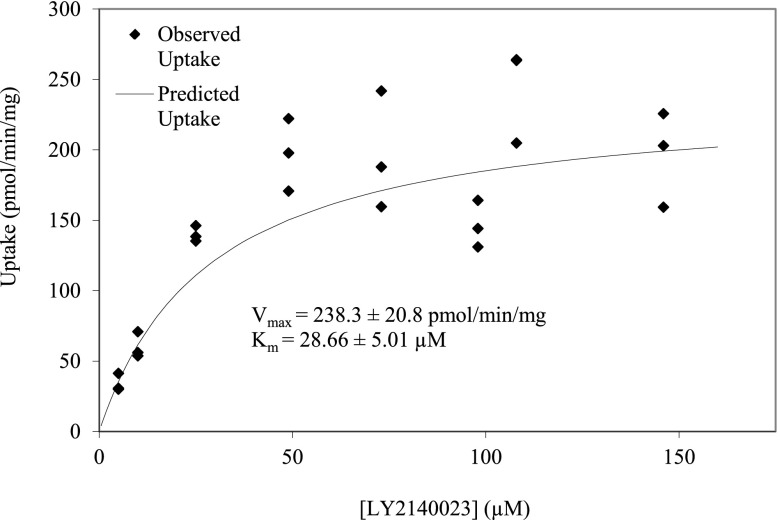
Concentration-dependent transport of LY2140023 in HeLa cells transiently transfected with PEPT1. The PEPT1-mediated transport of LY2140023 was observed over the concentration ranging from 5 to 146 *µ*M. The nonspecific accumulation of LY2140023 was measured by conducting a parallel experiment in HeLa cells transfected with an empty vector, and values were subtracted from the PEPT1 transfected uptake. The reactions were incubated for 3 minutes at room temperature. Each symbol represents the PEPT1-mediated transport of LY2140023 after nonspecific accumulation adjustment.

As illustrated in Fig. 3, the transport of the prodrug was a concentration-dependent process that followed Michaelis-Menten saturation kinetics. The nonspecific accumulation of the prodrug at each concentration was measured by conducting parallel experiments in HeLa cells transiently transfected with an pcDNA3.1 empty vector. After subtraction of nonspecific accumulation, the PEPT1-mediated transport of the prodrug was fitted to Michaelis-Menten kinetic model to obtain *K*_m_ and *V*_max_ values (Table 1).

**TABLE 1:** Concentration-dependent uptake of LY2140023 into HeLa cells to estimate kinetic parameters The concentration dependent transport of LY2140023 was evaluated from either 5 to 146 (experiment 1) or 6.4 to 149 μM (experiment 2) LY2140023. All concentrations were performed in triplicate. The estimated K_m_ and V_max_ values represent parameter ± S.E.M.

Experiment	*K*_m_	*V*_max_
	*μM*	*pmol/min/mg*
Experiment 1	28.66 ± 5.01	238.3 ± 20.8
Experiment 2	29.98 ± 5.29	572.8 ± 49.6

#### Determination of IC_50_ of known PEPT1 Inhibitors against the Prodrug.

The inhibitory potentials of known PEPT1 substrates on [^14^C]LY2140023 (prodrug) transport were determined using the dipeptide Gly-Sar as a positive control for inhibition of [^14^C]LY2140023 uptake. The potential to inhibit 10 *μ*M [^14^C]LY2140023 uptake was determined for several known PEPT1 substrates; the range of mean IC_50_ values from two separate experiments on [^14^C]LY2140023 uptake was between 0.46 and 25.90 mM (Table 2), with valacyclovir being the most potent and l-DOPA being the least potent inhibitor. A similar rank order of IC_50_ potency was observed for both [^14^C]Gly-Sar and the prodrug, with the exception of cephalexin being a more potent inhibitor of prodrug uptake than captopril, whereas the reverse is true on the inhibition of Gly-Sar uptake (Table 2). The inhibitory potency of LY2140023 against Gly-Sar was more potent than any tested drugs.

**TABLE 2:** Comparison of inhibitory potentials of known PEPT1 substrates on LY2140023 and Gly-Sar uptake

Compounds	IC_50_ against Gly-Sar^*A*^	IC_50_ against LY2140023	IC_50_ against LY2140023	Mean IC_50_ against LY2140023
	*mM*	*mM*	*mM*	*mM*
ALA	0.78 ± 0.26	1.77 ± 0.21	1.56 ± 0.22	1.67
Captopril	5.64 ± 0.81	13.45 ± 2.16	22.58 ± 3.26	18.02
Cefadroxil	1.88 ± 0.35	4.05 ± 0.44	5.93 ± 1.47	4.99
Cephalexin	11.1 ± 1.98	14.27 ± 3.23	12.95 ± 1.04	13.61
Enalapril	2.13 ± 0.36	10.97 ± 2.64	10.35 ± 0.42	10.66
Gly-Sar	N.A.	1.29 ± 0.26	0.69 ± 0.17	0.99
L- DOPA	14.9 ± 2.45	25.27 ± 2.57	26.52 ± 9.38	25.90
Valacyclovir	0.74^*B*^	0.41 ± 0.05	0.50 ± 0.11	0.46
LY2140023	0.018^*C*^			

^A^ Values of IC_50_ against Gly-Sar (50 *μ*M) were obtained from Zhang et al. (2004) except for valacyclovir.

^B^ The *K*_i_ value for valacyclovir was obtained from Ganapathy et al. (1998).

^C^ The mean IC_50_ values from two separate experiments, 0.023 ±0.09 and 0.013 ± 0.07 mM.

#### Evaluation of Clinical Implication using In vitro data.

To inform possible choices of inhibitors for a clinical study of PEPT1-mediated interactions, in vitro IC_50_ of five marketed drugs (cefadroxil, cephalexin, captopril, enalapril, and valacyclovir) were compared with their estimated concentrations in the gastrointestinal (GI) tract (I_2_). These concentrations (I_2_) were obtained by dividing the recommended clinical dose by a volume of 250 ml (volume of glass of water) (Table 3). The doses of compounds were obtained from Physicians' Desk Reference (1997). The ratios of I_2_/IC_50_ were substantially less than one (i.e., the estimated GI concentrations were lower than the mean in vitro IC_50_ values) for captopril (0.03 to 0.15), cephalexin (0.18 to 0.37), and enalapril (0.008 to 0.03), but were greater than one (i.e., the estimated GI concentrations were greater than the in vitro IC_50_ values) for cefadroxil (2.10 to 4.20) and valacyclovir (12.04 to 24.11), indicating the potential for an interaction. The I_2_/IC_50_ for LY2140023 using 80 mg dose was 48.5 greater than the ratios for valacyclovir.

**TABLE 3:** Potential for clinical DDI based on estimated concentrations in the GI tract

Compounds	Dose^*A*^	Estimated Intestinal Concentration^*B*^	Mean IC_50_ from Two in Vitro Experiments	I_2_/IC_50_
	*mg*	*mM*	*mM*	*mM*
Captopril	25 to 150	0.46 to 2.76	18.02	0.03 to 0.15
Cefadroxil	1000 to 2000	10.49 to 20.98	4.99	2.10 to 4.20
Cephalexin	250 to 500	2.48 to 4.98	13.61	0.18 to 0.37
Enalapril	10 to 40	0.08 to 0.32	10.66	0.008 to 0.03
Valacyclovir	500 to 1000	5.54 to 11.09	0.46	12.04 to 24.11
LY2140023	80	0.873	0.018	48.5

^A^ Dose obtained from Physicians' Desk Reference (1997) for oral administration.

^B^ Dose divided by 250 ml (glass of water) to estimate intestinal concentration of each compound.

### Clinical Results

#### Demographics and Disposition.

A total of 24 healthy subjects, 8 men and 16 women, with a mean age of 31.5 years (range 19 to 66 years of age) enrolled in this study. Twelve (50.0%) subjects were Caucasians, 11 ([45.8%) were African American, and 1 (4.2%) was Asian. The mean body mass index was 26.6 kg/m^2^.

Of the 24 subjects enrolled, 20 completed the study. The four subjects discontinued for the following reasons: failure to meet eligibility criteria (1 subject was discontinued before dosing), AE (1), and protocol violation Twenty-three subjects received 1000 mg valacyclovir alone, 21 subjects received 80 mg of LY2140023 alone, and 20 subjects received valacyclovir coadministered with LY2140023.

#### Pharmacokinetics.

After administration of the prodrug, the active moiety was formed rapidly and was present at the first sampling time in 18 of 21 subjects, which is consistent with previous clinical studies.

**Fig. 4.**
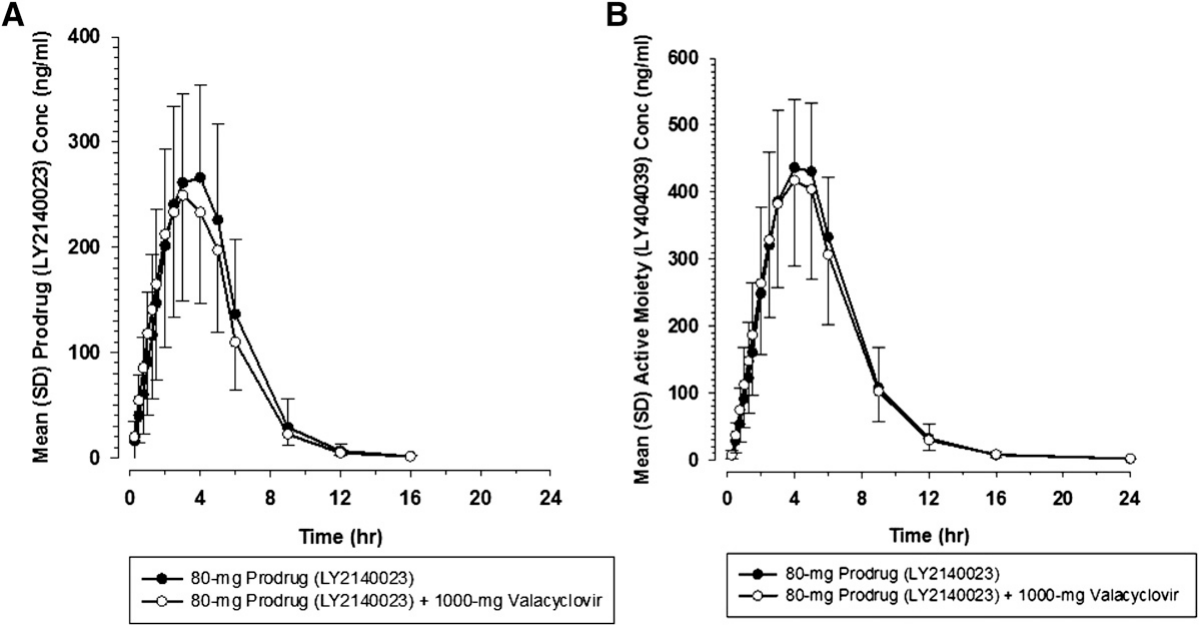
Mean LY2140023 (A) and mean LY404039 (B) concentration-time profile after a single 80-mg dose of LY2140023 administered alone (●) or with valacyclovir (○).

Mean PK parameters (Table 4) and profiles (Fig. 4A) were similar after dosing of LY2140023 (the prodrug) alone and when coadministered with valacyclovir. Ratios of LSM for *C*_max_ and AUC resulted in ratios that were close to 1 with confidence intervals contained within the 0.80 to 1.25 range (Table 5).

**TABLE 4:** Geometric mean of pharmacokinetic parameters for LY2140023 and LY404039 after a single 80-mg dose of LY2140023 administered alone or with valacyclovir Geometric mean (CV%) are presented for each parameter except where noted.

Treatment	LY2140023 Parameters		LY404039 Parameters	
	LY2140023 80 mg	LY2140023 80 mg + Valacyclovir 1000 mg	LY2140023 80 mg	LY2140023 80 mg + Valacyclovir 1000 mg
*N*	20	20	20	20
*C*_max_ (ng/ml)	307 (26)	272 (30)	477 (17)	444 (23)
t_max_^*a*^ (hour)	3.00 (2.00–5.03)	3.00 (1.25–6.00)	4.00 (3.00–6.07)	4.00 (2.50–6.00)
t_1/2_ (hour)	1.92 (26)	1.80 (24)	3.05 (14)	3.11 (16)
AUC(0-∞) (ng⋅h/ml)	1330 (27)	1210 (31)	2600 (16)	2490 (24)
CL/F (L/hour)	60.2 (27)	65.9 (31)	19.7 (16)	20.5 (24)
V_z_/F (l)	166 (27)	171 (21)	86.5 (24)	92.2 (34)
Fe			0.651 (15.9)	0.595 (13.2)
CLr (l/h)			12.8 (23.6)	12.2 (25.0)

^*a*^ Median (Min–Max)

**TABLE 5:** Statistical analysis of AUC and *C*_max_ for LY2140023, LY404039, valacyclovir, and acyclovir Geometric mean (CV%) are presented for each parameter except for t_max_, which presents Median (Min − Max).

Compound	Parameter	LY2140023 Alone (Reference)			LY2140023 + Valacyclovir (Test)			Ratio	90% CI
		*n*	LS Geometric Mean	90% CI	*n*	LS Geometric Mean	90% CI		
LY2140023	AUC(0-∞) (ng⋅h/ml)	20	1353.0	(1216.3, 1505.1)	20	1221.9	(1098.4, 1359.3)	0.90	(0.87, 0.94)
	*C*_max_ (ng/ml)	20	310.4	(279.9, 344.2)	20	275.0	(247.9, 304.9)	0.89	(0.83, 0.95)
LY404039	AUC(0-∞) (ng⋅h/mL)	20	2667.1	(2461.9, 2889.4)	20	2502.4	(2309.9, 2711.0)	0.94	(0.91, 0.97)
	*C*_max_ (ng/mL)	20	484.3	(447.8, 523.8)	20	447.1	(413.4, 483.6)	0.92	(0.87, 0.98)
		Valacyclovir Alone (Reference)			Valacyclovir + LY2140023 (Test)				
Valacyclovir	AUC(0-3) (ng⋅h/ml)	22	309.1	(251.1, 380.6)	20	277.5	(223.5, 344.6)	0.90	(0.71, 1.13)
	*C*_max_ (ng/ml)	23	245.4	(217.0,277.5)	20	258.3	(226.2, 294.8)	1.05	(0.89, 1.24)
Acyclovir	AUC(0-∞) (ng⋅h/ml)	23	17501.8	(16255.0, 18844.2)	20	17102.5	(15877.0, 18422.5)	0.98	(0.95, 1.00)
	*C*_max_ (ng/ml)	23	4397.9	(4025.2, 4805.0)	20	4604.4	(4190.1, 5059.6)	1.05	(0.94, 1.16)

Ratio is Test/Reference.

Similarly, the plasma PK parameters (Table 4) and profiles (Fig. 4B) for LY404039 (the active moiety) were similar after dosing of the prodrug alone and when coadministered with valacyclovir. Urinary excretion of the active moiety was also similar for the prodrug alone and with valacyclovir (Table 4), as measured by the fraction excreted (fe; 0.651 and 0.595, respectively) and renal clearance (CLr; 12.8 and 12.2 l/h, respectively). Ratios of LSM for *C*_max_ and AUC were also close to 1, and the CIs were contained within 0.80 and 1.25 (Table 5). The t_max_ analysis for prodrug and active moiety showed no significant differences observed for t_max_ (median of paired differences was 0.00 hour for prodrug and −0.07 hour for active moiety; Supplemental Table 1).

**Fig. 5.**
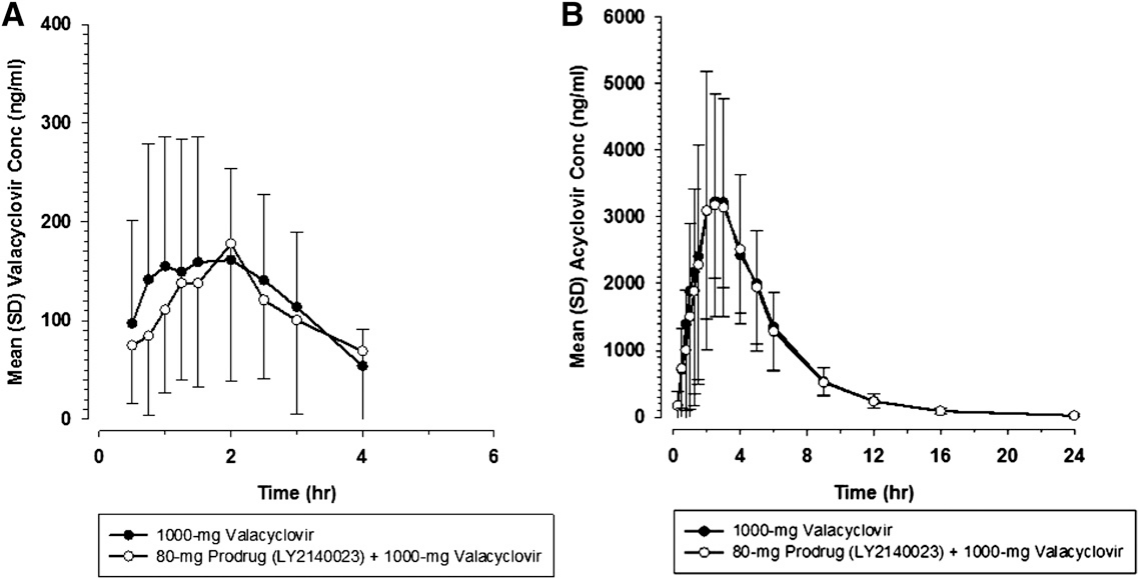
Mean valacyclovir (A) and acyclovir (B) concentration-time profiles after a single 1000-mg dose of valacyclovir administered alone (●) or with LY2140023 (○).

The valacyclovir plasma concentrations are limited and typically only measurable for 3 or 4 hours postdose (Phan et al., 2003), because conversion from valacyclovir (prodrug) to acyclovir (active metabolite) is rapid and efficient. Mean plasma profiles of valacyclovir were similar whether administered alone or with prodrug (Fig. 5A). As shown in Table 6, the valacyclovir plasma PK parameters and renal clearance were similar when administered alone or with the prodrug. Ratios of LSM for *C*_max_ and AUC were near 1 with CIs contained within the 0.80 to 1.25 range (Table 5), with the exception of the lower bound of the 90% CI for AUC(0–3), which was 0.71.

**TABLE 6:** Pharmacokinetic parameters for valacyclovir and acyclovir after a single 1000-mg dose of valacyclovir administered alone or with LY2140023 Geometric mean (CV%) are presented for each parameter except for t_max_, which presents Median (Min − Max).

Treatment	Valacyclovir Parameters		Acyclovir Parameters	
	Valacyclovir 1000 mg	LY2140023 80 mg + Valacyclovir 1000 mg	Valacyclovir 1000 mg	LY2140023 80 mg + Valacyclovir 1000 mg
N	23	20	23	20
*C*_max_ (ng/ml)	245 (41)	255 (29)	4400 (23)	4550 (28)
t_max_^*a*^ (hour)	2.00 (0.75–5.00)	2.00 (0.75–5.00)	2.50 (1.00–5.00)	2.51 (1.25–5.00)
t_1/2_ (hour)			3.70 (16)	3.63 (17)
AUC(0-3) (ng⋅h/ml)	311 (60)^*b*^	270 (67)		
AUC(0-∞) (ng⋅h/ml)			17,500 (20)	17,000 (23)
fe	0.00703 (64.7)	0.00776 (25.4)	0.460 (32.0)	0.488 (12.3)
CLr (l/h)	15.7 (79.5)	18.6 (23.4)	18.4 (42.0)	20.0 (24.7)

^*a*^ Median (Min–Max)

^*b*^ *n* = 22, Subject 108 not included as t_last_ was 2 hours.

After administration of the prodrug valacyclovir, the active metabolite acyclovir was formed rapidly. The acyclovir plasma PK and renal clearance were similar when administered alone or with the prodrug (Table 6). Similarly, the plasma profiles for acyclovir were similar with or without administration of the prodrug (Fig. 5B). The ratios of LSM for AUC and *C*_max_ were close to 1, and the 90% CI were within the 0.80 to 1.25 range for acyclovir (Table 5). The t_max_ analysis for valacyclovir and acyclovir showed no differences observed for t_max_ (median of paired differences was 0.00 hour for valacyclovir and 0.00 hour for acyclovir; Supplemental Table 1).

## Discussion

Despite broad substrate specificity, the potential for DDI at the PEPT1 transporter with new molecular entities (NMEs) has not been explored, because most of marketed PEPT1 substrates have low affinity for the transporter. However, the potential for an interaction cannot be ruled out when a drug or NME with a high inhibitory potential for PEPT1 is coadministered with a high-affinity substrate. Therefore, the current in vitro studies examined the DDI potential of LY2140023, a high-affinity substrate and a potent inhibitor of PEPT1, with PEPT1 substrates and inhibitors using transiently transfected HeLa cells. HeLa cells do not possess any detectable endogenous peptide transport activity and thus are ideal for overexpression of the peptide transporters (Sugawara et al., 2000). These in vitro results were then used to guide the design of the clinical drug-drug interaction study with valacyclovir.

As determined in this study, the uptake of LY2140023 (prodrug) was shown to be a PEPT1-mediated, proton-dependent process, whereas LY404039 (the active moiety) was not transported by PEPT1 and did not show proton dependency. The PEPT1-mediated uptake of prodrug was further confirmed in Pept1 knockout mice. In the Pept1 knockout mice, the exposure as in AUC_0–∞_ and *C*_max_ of the prodrug were only 10% of the exposure in the wildtype mice (data on file, Eli Lilly and Company). In addition, the passive diffusion of the active moiety was comparable in PEPT1 and pcDNA3.1 empty vector transfected cells, indicating low passive diffusion of this compound. The passive diffusion for the prodrug was also low, and its uptake into the PEPT1-transfected cells was primarily mediated by the transporter and not by passive diffusion, indicating the prodrug enters into cells mainly through an active uptake process. The low passive permeability of the prodrug and active moiety were confirmed in MDCK-MDR1 cells. In the MDCK-MDR1 cells, the passive permeability of the prodrug and active moiety were approximately 1 × 10^−6^ cm/s, which is lower than the low permeability marker atenolol (2.2 × 10^−6^ cm/s, data on file, Eli Lilly and Company) in this model. The MDCK cells have not shown to express PEPT1 (Quan et al., 2012), therefore the permeability represents their passive permeability across the monolayer. Taken together, the data clearly demonstrate that PEPT1 is responsible for the transport of the prodrug.

The inhibitory potential of LY2140023 was substantially more potent than any of the marketed drugs tested. As illustrated in Table 2, IC_50_ values of tested drugs against [^14^C]Gly-Sar ranged from 0.74 to 14.9 mM, whereas IC_50_ value for LY2140023 was 0.018 mM. Similarly, the affinity of the prodrug was substantially higher than the PEPT1 probe substrate Gly-Sar. The estimated *K*_m_ values of the prodrug in two separate experiments were 28.66 and 29.98 *μ*M, respectively (Table 1), whereas the estimated *K*_m_ value for Gly-Sar was 427.9 *μ*M (Supplemental Fig. S2).

A variety of structurally diverse, marketed drugs that are PEPT1 substrates with different affinity for the transporter (for review of PEPT1 substrates with different affinity, see Brandsch et al., 2008) were evaluated for their inhibitory potencies against the prodrug. The rank order of IC_50_ values of the tested drugs against the prodrug were similar to the rank order of IC_50_ values against Gly-Sar, indicating valacyclovir as the most and l-DOPA as the least potent inhibitor of PEPT1-mediated transport of the prodrug (Table 2). The possible clinical implication of PEPT1-mediated interactions was evaluated using the prodrug and a few well-characterized drug substrates of PEPT1: captopril, cefadroxil, cephalexin, enalapril, and valacyclovir. Subsequently, to normalize the inhibitory potential to the clinical dose, the in vitro IC_50_ values were divided by 250 ml (the volume of a glass of water). This is adapted from the transporter guidelines published by the US Food and Drug Administration (http://www.fda.gov/downloads/drugs/guidancecomplianceregulatoryinformation/guidances/ucm292362.pdf), European Medicines Agency (http://www.ema.europa.eu/docs/en_GB/document_library/Scientific_guideline/2012/07/WC500129606.pdf), and the International Transport Consortium (Giacomini et al., 2010) for P-glycoprotein and BCRP intestinal interaction. The guidelines suggest that the in vitro IC_50_ values for drugs be compared with the estimated concentrations in the gastrointestinal (GI) tract (I_2_), which were obtained by dividing the recommended clinical dose by a volume of 250 ml (volume of a glass of water). The guidelines recommend conducting an in vivo DDI study when I_2_/IC_50_ is greater than 10 (Giacomini et al., 2010; http://www.fda.gov/downloads/drugs/guidancecomplianceregulatoryinformation/guidances/ucm292362.pdf; http://www.ema.europa.eu/docs/en_GB/document_library/Scientific_guideline/2012/07/WC500129606.pdf). For this study, because there is no prior experience to define a cut off value for the ratio, the inhibitor that gave the greatest I_2_/IC_50_ ratio was chosen for the clinical study. Table 3 shows that the ratios of I_2_/IC_50_ were substantially less than 10 (i.e., estimated GI concentrations were lower than the in vitro IC_50_ values) for captopril, cephalexin, and enalapril, between 1 and 10 for cefadroxil, and greater than 10 for valacyclovir, suggesting the highest potential for clinical interaction for valacyclovir. The ratio for LY2140023 was close to 50, indicating potential for interaction for the prodrug following the guidelines.

Subsequently, a clinical study was designed to evaluate LY2140023 as both a substrate and an inhibitor of PEPT1. The coadministration of LY2140023 and valacyclovir did not affect the PK of each other or their respective active moieties (LY404039 or acyclovir), indicating no clinical DDI between the prodrug and valacyclovir. The data also showed that the presence of the prodrug or valacyclovir did not affect the conversion of prodrug to its active moiety for either LY2140023 or for valacyclovir. The lack of interaction on the conversion of the prodrug and valacyclovir to its corresponding active moieties was expected, because different enzymes are responsible for their activation. Dehydropeptidase 1 has been shown to cleave the prodrug to its active moiety (Moulton et al., 2015) and valacyclovirase (biphenyl hydrolase-like protein) to cleave valacyclovir to acyclovir (Marsillach et al., 2014). For both active moieties there was no change in the CL/F or the CLr, indicating that coadministration of the drugs did not affect the renal clearance of each other. Furthermore, if there is a weak interaction at PEPT1, a shift in T_max_ values may be observed. However, no shift in T_max_ was observed for any of the entities studied. Also, for valacyclovir there was no significant change in the AUC(0–3 hours), again indicating no interaction at PEPT1.

In this study, we illustrated how in vitro studies can guide the design of clinical DDI studies for transporter-based interactions. In vitro screening of inhibitory potencies of multiple drugs that compete at the transporter could give the rank order of inhibitory potencies and an analysis for potential for DDI in relation to oral dose of the compound. Therefore, unnecessary in vivo studies could be avoided, while focusing on the most relevant potential for DDI. Although the in vitro study indicated the potential for a DDI between the prodrug and valacyclovir according to guideline for other intestinal transporters, an in vivo DDI study showed no interaction of these two drugs via PEPT1. Therefore, our results clearly illustrated that a clinical DDI at PEPT1 is highly unlikely even with a NME with high affinity for the transporter.

## Abbreviations

AE: adverse event
ALA: 5-aminolevulinic acid
AUC: area under the curve
BCRP: breast cancer resistance protein
CI: confidence interval
*C*_max_: maximum concentration
DDI: drug-drug interaction
DOPA: dihydroxyphenylalanine
GI: gastrointestinal
Gly-Sar: glycylsarcosine
LLQ: lower limit of quantification
SM: least squares means
mGlu2/3: metabotropic glutamate 2/3
NMEs: new molecular entities
PEPT1: peptide transporter 1
P-gp: P-glycoprotein
PK: pharmacokinetics
SM: least squares means
ULQ: upper limit of quantification
